# Prescribing practices of Hormone Therapy for Menopausal females by OBGYN of Pakistan

**DOI:** 10.12669/pjms.39.1.6176

**Published:** 2023

**Authors:** Nighat Shah, Hira Tariq, Farah Khan, Nusrat Shah

**Affiliations:** 1 Dr. Nighat Shah, Associate Professor, Sindh Reproductive and Genetic Health Center, Jinnah Sindh Medical University, Karachi, Pakistan; 2 Dr. Hira Tariq, Lecturer, APPNA Institute of Public Health, Jinnah Sindh Medical University, Karachi, Pakistan; 3 Dr. Farah Khan, Senior Instructor, Sindh Reproductive and Genetic Health Center, Jinnah Sindh Medical University, Karachi, Pakistan; 4 Dr. Nusrat Shah, Professor & Head of Department of Obstetrics & Gynecology, Dow University of Medical Sciences, Karachi, Pakistan

**Keywords:** Hormonal treatment, Menopause, Indications, Barriers

## Abstract

**Objectives:**

To assess the prescribing practice of obstetricians and gynecologists (OBGYN) regarding Menopausal Hormone therapy (MHT) for menopausal females and assess the knowledge regarding indications for prescribing MHT and identify the barriers to HRT.

**Methods:**

This was a cross-sectional study conducted from May 2021 to December 2021. The participants were OBGYN experts (MCPS, FCPS, MRCOG) and senior experts. The tool was formulated after looking at contemporary literature and then validated by experts for face, content and construct validity. It was piloted and hence fourth given to study participants after approval by IRB of JSMU. Data was analyzed by SPSS version 22. Mean and Standard Deviation of categories in Likert scale were calculated.

**Results:**

Majority of the participant gynecologists prescribed HRT for Hot flushes and vaginal dryness having the highest mean scores nearing 4 on a Likert scale of (1-5). The prescription for other menopausal symptoms was observed to be less. The highest score for category of women in whom hormone therapy is specifically justified was “Premature ovarian failure” (4.37) followed by “Hysterectomy with bilateral oophorectomy before the age of 50” (4.23).

**Conclusion:**

Pakistani gynecologists are more cautious in their management strategies concerning MHT. Most of the gynecologists showed good and up to date information while prescribing MHT however knowledge for preventing fractures, alleviating anxiety/depression and weight gain was less among the gynecologist of Pakistan. We recommend refresher courses and online webinars for updated information on menopause and its management.

## INTRODUCTION

Menopause is natural phase which designates an end of ovarian function and hence an end of women s capacity to reproduce. Biological menopause age is usually around 45–55 year.^1^ Menopausal hormonal changes are known to have symptoms like vasomotor symptoms, urogenital, psychological related to moods, sleep and loss of libido. Long term effects include osteoporosis, coronary heart disease, memory and dementia issues^1^. Additionally, women may experience debilitating effects which may significantly impair quality of life and have long term health consequence. As the age expectancy improves more and more women will fall in this age bracket.^2^,^3^ Studies have shown that quality of life deteriorates and menopausal hormonal therapy (MHT) significantly improves symptoms although it’s use is very limited in Pakistani society.^3^

Evidence regarding use of MHT and prescribing practices have gone through conflicts and dilemmas. In seventies estrogen only was preferred, the risk of endometrial cancer then made the practitioners to add progesterone.^4^ Women health initiative (WHI) was the first randomized control trial study on risks and benefits of MHT.^5^ This trial showed the risk of breast cancer, coronary heart disease, stroke and venous thromboembolism associated with MHT which was latter reanalyzed and confirmed by British Million Women Study (WMI).^6^ The prescribing practice of MHT suffered a huge setback.^4^

Henceforth many studies and even review studies of WHI showed benefits of hormonal treatment in terms of improvement in vasomotor symptoms and prevention of osteoporosis. Many authors recommend practice should be guided by local context and strong evidence.^4^ The current practices are guided by factors such as physician knowledge^7^, their own menopausal status and geographical location including acceptance as per social norms.^8^

Pakistan studies show that most providers are not expert gynecologists, and hence are unaware about potential benefits and risks of MHT. Therefore, are unlikely to recommend it, whereas evidence demonstrates 75% women may consider MHT if recommended by their care providers.^9^

The women are increasingly aware regarding knowledge of menopause but still practicing gynecologists are cautious in prescribing medications for its treatment. The approach towards management is influenced by the cultural, social and religious beliefs and that creates a great impact on the practices of the local gynecologists of the region.^10^ Many studies have been performed to show the trend of using MHT in general population and prescription by physicians all over the world.^10^ Only a few studies have explored gynecologists personal use and attitude towards and our MHT study is one of a kind which provides insight on “what should be” and “what is actually done” in our region. The main objectives of this study were to assess the prescribing practice of obstetricians and gynecologists (OBGYN) regarding MHT for menopausal females and assess the knowledge regarding indications for prescribing MHT and identify the barriers to MHT.

## METHODS

This was a cross sectional study conducted in Karachi. The practicing gynecologists (MCPS, FCPS or MRCOG), senior trainees, of tertiary care hospitals were approached through emails, WhatsApp and personally in conferences and OBGYN seminars. Sample size was calculated by using statistical software “Open Epi”. Expecting a frequency of 19% for prescription of HRT (Asian Menopause survey)^11^ and total number of fellows and members of CPSP as 5611, the sample size came out to be 227, at confidence level of 95% and bound of error of 5%.

Questionnaire was formulated by experts after looking at relevant contemporary literature, and research. It was then corrected by technical and content experts of reproductive health and piloted for comprehensiveness, time and cultural constraints if any. Section-A consisted of information regarding socio-demographics and work variables and Section-B contained questions on knowledge, practices and barriers related to MHT. Almost all questions were based on Likert scale. Data collectors were properly trained prior to data collection by the Principle Investigator. Data was collected after IRB approval from May 2021 to December 2021 by experienced data collectors from medical field and supervised by the Principle investigator.

Data was analyzed using SPSS version 22, software. Mean and Standard deviation of the categories in Likert scale were calculated. Written Informed consent was sought from all participants. The ethical approval was sought from IRB (Institutional Review Board) of Jinnah Sindh Medical University (JSMU-IRB-2017-75).

## RESULTS

More than half of the participants were 30-40 years of age 109 (58.9%) with majority being females179 (96.8%) and belonging to Sindh 154 (83.2%). Majority practiced in Urban areas 126 (68.1%) with more than half working in private sector 101 (54.6%). Nearly one third of the participants have been practicing for 20-40 years 48 (25.9%) having an OPD of 50-100 patients daily. Nearly one quarter 43(23.2%) saw 21-40 menopausal patients in a month and majority 125 (67.6%) prescribed less than 10 patients with hormone treatment (Table-I).

**Table-I:** Socio-demographic and work characteristics of the study participants (n=185).

Variables	N %
***Age***	
30-40	109(58.9%)
41-50	38 (20.5%)
51-60	26 (14.1%)
61-70	12 (6.5%)
***Gender***	
Male	6(3.2%)
Female	179 (96.8%)
***Ethnicity***	
Sindh	154 (83.2%)
Punjab	15 (8.1%)
KPK	11 (5.9%)
Baluchistan	5 (2.7%)
***Practice setting***	
Rural	59 (31.9%)
Urban	126 (68.1%)
***Institute***	
Private	101 (54.6%)
Government	69 (37.3%)
Others	15 (8.1%)
***No. Of Year Since Practice***	
>20 years	133 (71.9%)
20-40	48 (25.9%)
40-60	4 (2.2%)
***No. Of Patient on Daily Basis***	
Less than 50	115 (62.2%)
51-100	57 (30.8%)
More than 100	13 (7%)
***No of Menopausal patients seen in a month***	
Less than 20	135 (73%)
21-40	43 (23.2%)
41-60	7 (3.8%)
***Prescribe Hormones to Menopausal women***	
Yes	135(73%)
No	50 (27%)
***No of Patients prescribed hormonal treatment***	
Less than 10	125 (67.6%)
11-20	60 (32.4%)

**Fig.1:**
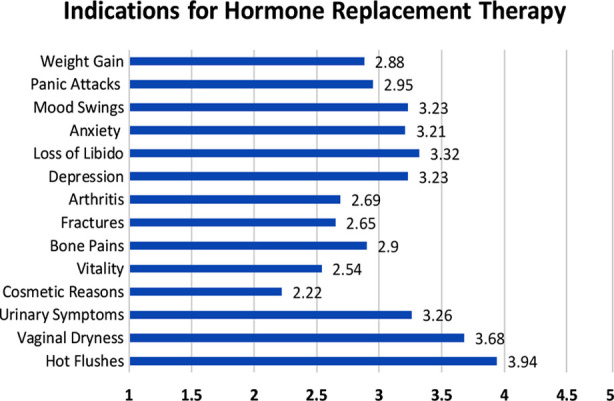
Mean scores for prescription of Hormonal Treatment for most common symptoms on a scale from 1 (Never prescribed) to 5 (Always prescribed).

When indication of prescribing menopausal hormonal therapy was inquired, (Fig.1), the highest score for prescription was on the symptom of “Hot Flushes” (3.94) followed by “Vaginal Dryness” (3.68) “Loss of Libido” (3.32) and “Mood Swings” (3.23) on a scale of one to five, where one denotes strongly disagree and five strongly agree.

**Fig.2:**
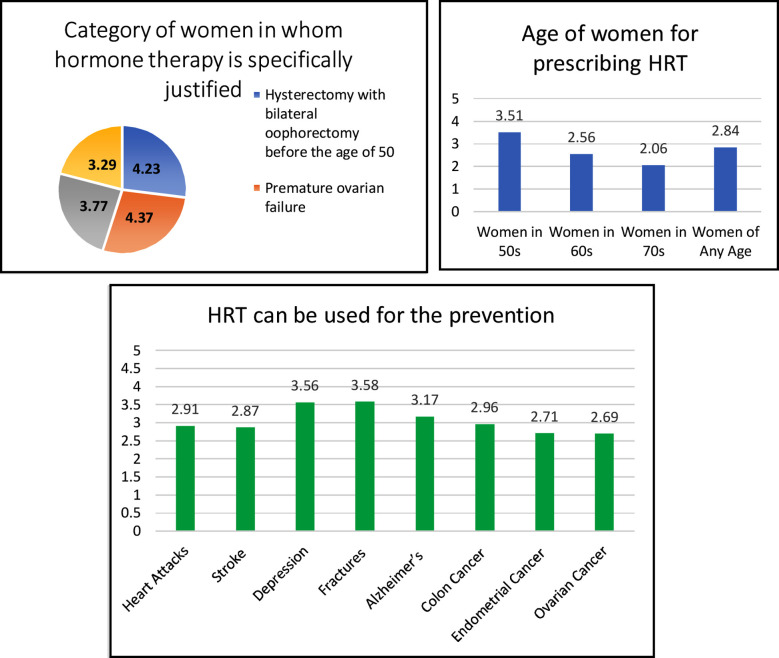
Knowledge of Hormone Replacement Therapy among Study Participants.

Most obgyn experts agreed that hormone therapy is specifically justified in “Premature ovarian failure” (4.37) followed by “Hysterectomy with bilateral oophorectomy before the age of 50” (4.23). The finding of Prescription according to the age of women showed the highest score for “women in 50s” (3.51) followed by “women of any age” with a mean score of agreement (2.84). For the question of hormone treatment can be used to prevent certain conditions, the highest score was for “Fractures” (3.58) followed by “Depression” (3.56) and Alzheimer’s (3.17). (Fig.2)

Amongst our participants the fear of breast cancer and endometrial cancer were main deterrents for prescribing hormonal treatment. The findings of controversies regarding MHT on a scale of one (strongly disagree) to five (strongly agree) showed highest mean value for “breast cancer” (3.89 ± 1.37) followed by “endometrial cancer” (3.63 ± 1.49).

The “cost” (4.14 ± 1.27) of treatment was the major hurdle n prescribing MHT followed by the “Side Effects” (3.70 ±1.21) and “Religious barriers” (2.95 ± 1.31) on a scale of one (strongly disagree) to five (strongly agree) (Table-II).

**Table-II:** Controversies surrounding menopausal hormonal treatment and Barriers in prescribing HRT.

Variables	Mean and Standard deviation
***Controversies***	
Breast Cancer	3.89 ± 1.37
Endometrial Cancer	3.63 ± 1.49
Weight gain	3.38 ± 1.33
Abnormal bleeding	3.17 ± 1.32
***Barriers***	
Cost	4.14 ± 1.27
Side effects	3.70 ±1.21
Limited research	2.78 ± 1.33
Controversies of WHI study	2.89 ± 1.37
Culture	3.14 ± 1.33
Religion	2.95 ± 1.31

## DISCUSSION

In our study most of the respondents showed good knowledge regarding alleviation of symptoms by MHT which included hot flushes, vaginal dryness, and loss of libido and mood swings. This was similar to a study conducted in China where ob/gyn practitioners showed major prescribing practices for hot flushes and for fracture prevention,^12^ our study population however showed a smaller number of gynecologists prescribe MHT for fracture prevention.

Evidence has supported that MHT especially a combined one helps in redistribution of fat after menopause and decrease in BMI.^13^ Our study showed less knowledge regarding obesity and HRT and majority of the gynecologists believe that MHT is a threat to weight gain. Although the changes in the hormonal milieu, chronological aging of women with decline in physical activity, dietary patterns and recurrent emotional eating episodes together with psychological distress also contributes to the increase in total body fat and waist circumference in menopausal women.^13^

According to our survey majority of the practicing gynecologists strongly agree in prescribing MHT after premature surgical menopause. This is similar to practices of ob/gyn all over the world that are in favor of prescribing MHT after hysterectomy and bilateral salpingo-ophorectomy especially in a younger age group^14^.A good number of the participants agreed on treatment for Premature ovarian failure. It is important to understand that they have unique needs and require special attention.^15^ Long term results are better when MHT is prescribed for patients with chromosomal diseases.^16^

Less than half of the practicing doctors of our group prescribed MHT for prevention of fractures. Osteoporosis and the rate of bone loss increases significantly in menopausal women ultimately increasing their risk to bone fractures.^17^ The benefits of estrogen as a treatment for osteoporosis have been demonstrated.^17^,^18^ Also, small number of the participants believe that hormonal therapy is preventive against heart attacks and stroke. Randomized clinical trials and observational data provide evidence that estrogen-containing MHT may decrease coronary heart disease and mortality in women younger than 60 years of age and within 10 years of menopause.^7^,^19^ More educational sessions are needed to make our practicing doctors aware regarding their practices for prevention of cardiac events.

Most of the specialists agreed that it helps in fighting depression. Several studies have also confirmed the role of MHT in fighting depression.^20^ The participants of our study showed impartial opinion regarding treatment with MHT and their protective role in endometrial, ovarian and colon cancer. Data available at the moment do not allow discriminating for dose, routes of administration, and bioavailability and tissue effects of different compounds. A study in Karachi on Type-I and Type-II endometrial cancers found only one patient of Type-II on MHT but no Type-I patient on MHT despite previous studies indicating MHT association with Endometrial cancers.^21^

Considering Menopause, a disease entity and giving treatment has its own role in terms of cost which was strongly supported as a restriction by our gynecologists who are practicing in a developing country. Similar finding of cost of treatment, lack of awareness and controversies regarding MHT was also highlighted in a study from India reflecting patient perspectives.^22^-^23^ Previous literature from Pakistan however highlighted lack of awareness among gynecologists/obstetricians as a major barrier which is in contrast to our study.^24^ The results of WHI trial negatively influenced the practices of prescribing MHT which was another barrier identified.^24^ Cultural and religious believes were also identified by our doctors as a barrier to prescribe this medicine for relief. This finding was also consistent with previous literature.^23^

When the prescription for MHT is required a decision to choose the right patient requires a personalized discussion regarding the balance of potential risks and benefits as individualized to that woman’s health circumstances.^24^ Important factors to take into consideration include the woman’s age, type and timing of menopause, impact of symptoms on quality of life, health history, family medical history, and personal preferences.^25^ The balance of benefits and risks for MHT is most favorable within the first ten years of menopause, or up to around 60 years of age. During this window of opportunity, estrogen-containing hormone therapy not only relieves menopausal symptoms for women at low risk, but also may have a positive impact on women’s cardiovascular and bone health. This was reflected in the knowledge of our practicing gynecologists when prescribing HRT.

### Strengths:

The strength of the study was the consistent data collected by Principal Investigator and the team of data collectors on platforms like conferences and academic activities where wide geographical coverage of participants is seen. As our study was based on routinely collected data, it was not susceptible to recall bias.

### Limitations:

It include lack of completion of study sample which was 227 and we managed to gather data of 185 participants due to the busy schedule of gynecologists while half-filled forms were not included in the analysis.

## CONCLUSION

Our study showed Pakistani gynecologists are more cautious in their management strategies concerning MHT probably influenced by the results of WHI trial. Most of the gynecologists showed good and up to date information while prescribing MHT however knowledge for preventing fractures, depression and weight gain was less among the gynecologist of Pakistan.

### Recommendations:

We recommend refresher courses and online webinars for updated information on menopause and its management. Campaigns on social media platforms will also play a role.

### Authors’ Contribution:

**NS:** Concept of study, design, methodology and data collection, responsible and accountable for the accuracy and integrity of the work. **HT:** Data Collection, Data Analysis, and Manuscript Review and responsible and accountable for the accuracy and integrity of the work. **FK:** Data collection, literature search and review. **NS:** Finalized the concept, helped in design and expert review of manuscript.

## References

[1] Kalra B, Kalra S (2020). Menopause:A Matter of Good Health. J Pak Med Assoc.

[2] Shorey S, Ng ED (2019). The experiences and needs of Asian women experiencing menopausal symptoms:A meta-synthesis. Menopause.

[3] Khokhar S (2013). Knowledge, attitude and experience of menopause. Pak J Med Res.

[4] Fait T (2019). Menopause hormone therapy:Latest developments and clinical practice. Drugs Context.

[5] Manson JE, Chlebowski RT, Stefanick ML, Aragaki AK, Rossouw JE, Prentice RL (2013). Menopausal hormone therapy and health outcomes during the intervention and extended poststopping phases of the Women's Health Initiative randomized trials. JAMA.

[6] Colau JC, Vincent S, Marijnen P, Allaert FA (2012). Efficacy of a non-hormonal treatment, BRN-01, on menopausal hot flashes. Drugs R&D.

[7] Taylor HS, Kagan R, Altomare CJ, Cort S, Bushmakin AG, Abraham L (2017). Knowledge of clinical trials regarding hormone therapy and likelihood of prescribing hormone therapy. Menopause.

[8] Vallejo M, Witis S, Ojeda E, Mostajo D, Morera F, Meruvia N (2016). Does the menopausal status of female gynecologists affect their prescription of menopausal hormone therapy?. Climacteric.

[9] Mallhi TH, Khan YH, Khan AH, Mahmood Q, Khalid SH, Saleem M (2018). Managing hot flushes in menopausal women:A review. J Coll Physicians Surg Pak.

[10] Mackey S, Teo SSH, Dramusic V, Lee HK, Boughton M (2014). Knowledge, attitudes, and practices associated with menopause:A multi-ethnic, qualitative study in Singapore. Health Care Women Int.

[11] Huang KE, Xu L, Nasri N, Jaisamrarn U (2010). The Asian Menopause Survey:knowledge, perceptions, hormone treatment and sexual function. Maturitas.

[12] Wang Y, Yang X, Li X, He X, Zhao Y (2014). Knowledge and personal use of menopausal hormone therapy among Chinese obstetrician-gynecologists:results of a survey. Menopause.

[13] Chopra S, Sharma KA, Ranjan P, Malhotra A, Vikram NK, Kumari A (2019). Weight management module for perimenopausal women:A practical guide for gynecologists. J Mid-Life Health.

[14] Garg N, Behbehani S, Kosiorek H, Wasson M (2020). Hormone replacement therapy prescription after premature surgical menopause. J Minim Invasive Gynecol.

[15] Panay N, Kalu E (2009). Management of premature ovarian failure. Best Pract Res Clin Obstet Gynaecol.

[16] Cleemann L, Hjerrild BE, Lauridsen AL, Heickendorff L, Christiansen JS, Mosekilde L (2009). Long-term hormone replacement therapy preserves bone mineral density in Turner syndrome. Euro J Endocrinol.

[17] Tariq S, Lone KP, Tariq S (2016). Comparison of parameters of bone profile and homocysteine in physically active and non-active postmenopausal females. Pak J Med Sci.

[18] Levin V, Jiang X, Kagan R (2018). Estrogen therapy for osteoporosis in the modern era. Osteoporos Int.

[19] Lobo RA (2013). Where are we 10 years after the Women's Health Initiative?. J Clin Endocrinol Metabol.

[20] Rubinow DR, Johnson SL, Schmidt PJ, Girdler S, Gaynes B (2015). Efficacy of estradiol in perimenopausal depression:So much promise and so few answers. Depress Anxiety.

[21] Malik TY, Chishti U, Aziz AB, Sheikh I (2016). Comparison of risk factors and survival of type 1 and type II endometrial cancers. Pak J Med Sci.

[22] Hillman S, Shantikumar S, Ridha A, Todkill D, Dale J (2020). Socioeconomic status and HRT prescribing:a study of practice-level data in England. Br J Gen Pract.

[23] Singh N, Chouhan Y, Patel S (2018). Treatment seeking behavior among post-menopausal females attending out-patient clinic of Obstetrics and Gynecology at a tertiary care centre, Bhopal, Madhya Pradesh. Int J Community Med Public Health.

[24] Kalra B, Lathia T, Kalra S, Malhotra N (2020). Barriers and bridges in menopause hormonal therapy. J Pak Med Assoc.

[25] Sood R, Faubion SS, Kuhle CL, Thielen JM, Shuster LT (2014). Prescribing menopausal hormone therapy:An evidence-based approach. Int J Women's Health.

